# An Analysis of the Educational and Health-Related Benefits of Nature-Based Environmental Education in Low-Income Black and Hispanic Children

**DOI:** 10.1089/heq.2019.0118

**Published:** 2020-05-18

**Authors:** Nadav Sprague, David Berrigan, Christine C. Ekenga

**Affiliations:** ^1^ Brown School, Washington University in St. Louis, St. Louis, Missouri, USA.; ^2^ Division of Cancer Control and Population Sciences, National Cancer Institute, Bethesda, Maryland, USA.

**Keywords:** health-related quality of life, nature contact, environmental education, sustainability, youth, environmental justice

## Abstract

**Background:** Low-income and non-white children experience disparities in health, education, and access to nature. These health disparities are often associated and exacerbated by inequities in the U.S. educational system. Recent research suggests that nature contact may reduce these health and educational disparities for urban low-income populations. Nature-based education (NBE) uses nature contact to inspire curiosity and improve health. This study examines the health and educational outcomes of a 15-week NBE intervention for urban low-income, black and Hispanic children 10–15 years of age.

**Methods:** Children (*n*=122) completed a pre-intervention and post-intervention survey that addressed seven science, technology, engineering, and math (STEM)-capacity items (leadership, teamwork, science relevance, sustainability relevance, STEM self-efficacy, science interest, and overall STEM capacity) and six widely used health-related quality-of-life (HRQoL) domains (physical health functioning, emotional health functioning, school functioning, social functioning, family functioning, and overall HRQoL). Focus groups with participating students and post-intervention surveys of NBE mentors and teachers explored perceptions of the intervention impact.

**Results:** There were statistically significant positive changes in STEM capacity and HRQoL for participating students. For example, children's overall STEM capacity and overall HRQoL scores improved by 44% and 46%, respectively (both *p*<0.05). Qualitative data highlighted the intervention's educational and health benefits.

**Conclusions:** These results support further research quantifying the effects of NBE on STEM capacity and HRQoL in urban, low-income, black and Hispanic children.

## Introduction

Across the health spectrum, non-white and low-income children experience health disparities.^1^ These childhood health disparities involve differences in health and behavioral outcomes for specific racial, ethnic, and socioeconomic populations.^1^ Childhood health disparities have both immediate and long-term consequences.^2^ These potentially lifelong consequences include increased risks for long-term adverse health outcomes (e.g., diabetes, cardiovascular disease, and cancer)^3–7^ as well as establishment of negative health behaviors at an early age, which often continue into adulthood.^8,9^ Black and Hispanic children as well as low-income children also face educational inequalities compared to white or high-income children.^10,11^ Furthermore, education is an upstream social determinate of health, and programs that reduce educational inequities promote health equity.^12^ There are significant racial and socioeconomic disparities in access to and contact with nature.^13–16^ White children have significantly more nature contact than black children.^17^ Recent attention has focused on the potential role of nature contact as an influence on health disparities for urban low-income populations.^15^

There is a growing body of evidence that suggests that nature contact is a practical method for promoting better physical, emotional, mental, and overall health for children as young as 10 years.^18–21^ Childhood nature contact promotes positive youth development, improved cognition, childhood resilience, and reduction of mental health disorders (e.g., anxiety, depression, and attention deficit hyperactivity disorder [ADHD]).^22–26^ Both cross-sectional and prospective cohort studies have found that increased childhood nature contact is positively associated with increased physical activity.^21,27–29^ Furthermore, a few studies have found greater physical and mental health benefits from green exercise (outdoor physical activities in natural settings) than traditional forms of exercise.^30–33^

### Nature-based education

**FIG. 1.**
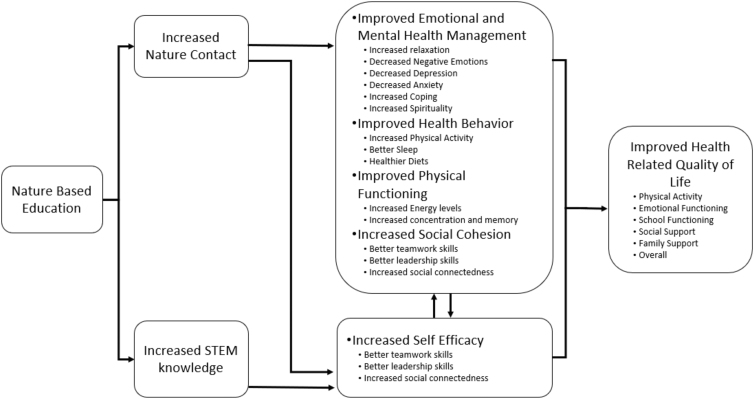
Conceptual model of NBE impacts on HRQoL and STEM knowledge. HRQoL, health-related quality of life; NBE, nature-based education; STEM, science, technology, engineering, and math.

Nature-based education (NBE) is a form of environmental education that uses nature contact to increase environmental awareness and inspire curiosity.^29,33^ Our conceptual model of NBE's impacts on health-related quality of life (HRQoL) and science, technology, engineering, and math (STEM) knowledge (Fig. 1) represents a synthesis of the emerging research on environmental education and pathways through which it might benefit children,^34^ along with potential pathways through which nature contact may promote positive health outcomes.^29,35,36^ In this model, NBE may increase STEM knowledge, which is associated with increased self-esteem and family cohesion for low-income and non-white children^37,38^ NBE interventions may improve children's academic success, which influences childhood HRQoL.^39,40^ In addition, NBE incorporates nature-contact components, increasing the frequency of nature contact and its benefits.^24,29,36,41^

A pilot study found that this NBE intervention significantly improved overall HRQoL scores and family support HRQoL domain scores for low-income, black and Hispanic children in St. Louis, Missouri.^42^ In this study, we investigate how NBE influences health and educational outcomes for low-income, black and Hispanic children in St. Louis, MO, USA. This study includes a larger sample of students, a further examination of STEM-capacity, and qualitative aspects of the response to the NBE intervention in students, teachers, and NBE mentors. Our study tests the hypothesis that an NBE intervention improves two outcomes: (1) HRQoL and (2) STEM knowledge and self-efficacy (STEM capacity).

## Methods

### NBE intervention

A NBE intervention was developed for low-income black and Hispanic children in St. Louis, Missouri. The intervention was administered to three elementary and middle schools in the St. Louis Public Schools District (SLPS) during the 2018–2019 academic year. SLPS is the largest urban public school district in the St. Louis metropolitan area.^43^ In the 2018–2019 school year, SLPS had an enrollment of 20,879 students; 80% of whom were black and 4% Hispanic.^43^ The SLPS district faces well-documented and significant educational inequalities compared to neighboring school districts.^44–46^ For example, in the 2018 statewide standardized assessment, only 23% of SLPS students scored proficient or advanced in the English and Language Arts section and only 19% scored proficient or advanced on the Math section compared to the greater St. Louis metropolitan area averages of 47% and 41%, respectively.^43^ To qualify for the NBE intervention, schools were required to have more than 95% of the student body eligible for free or reduced meals through the National School Lunch Program and the School Breakfast Program.

The NBE intervention was facilitated by Gateway to the Great Outdoors, a regional nonprofit organization founded by one of the investigators (N.S.).^47^ The NBE intervention consisted of weekly STEM-based environmental education classroom lessons and monthly nature-based outdoor field trips facilitated by volunteer undergraduate mentors (NBE mentors). The NBE mentors were recruited, trained, and overseen by the nonprofit organization. Every week, the NBE mentors would visit their assigned SLPS classroom and teach interactive lessons created by the nonprofit organization, based on the classroom's STEM curricula, the state of Missouri's Testing Standards,^48^ EPA lesson plans,^49^ Next Generation Science Standards,^50^ and other environmental science courses. The nature-based outdoor field trips were created to provide context for classroom activities and reinforce classroom learning. In conjunction, the weekly in-class environmental education lessons and monthly nature-based field trips were developed to promote teamwork, leadership, environmental and conservation awareness, STEM knowledge, and improved HRQoL, while also teaching outdoor skills and changing SLPS students' perceptions of science and sustainability. Table 1 provides an overview of the interventions' curricula, monthly field trips, and learning objectives.

**Table 1.** Nature-Based Education Intervention's In-Class Curriculum, Learning Objectives, and Field Trip Schedule for 2018–2019 School Year

	Semester 1		Semester 2	
Week	In-class lesson	Learning objectives	In-class lesson	Learning objectives
1	Introductions and Planting	Introducing the Nature-Based Education intervention. Students will then meet in their mentoring group and play fun ice-breaker activities. Each mentoring group will plant vegetable seeds that they will grow and harvest throughout the semester.	Natural Disasters	Students will learn about various natural disasters and will discuss the ways natural disasters have affected their community and the world.
2	DO NOT TRASH IT: Reduce, Reuse, and Recycle!	Students will learn about the difference between recycling and trash. They will explore the different ways people reuse “trash” creatively through an art project and create recycling programs at their schools if the schools do not currently have. Older students will learn about the science behind reducing our waste and efforts being made toward zero-waste products.	Pollution	Students will investigate the different forms of pollution and dive deeper into studying water pollution.
3	LNT	Students will learn about the camping LNT principals.	Weather vs. Climate	Students will understand the difference between weather and climate. Then they will begin to discuss climate change and apply prior knowledge from the past week's lesson on pollution.
4	Green City	Students will explore the different ways that their communities and the world can be more “green” or environmentally friendly.	Renewable Energy vs. Nonrenewable Energy	Students will compare and contrast renewable energy and nonrenewable energy.
5	Scientists	Students will learn about different important Scientists, focusing on scientists of color. Students will choose an environmental issue to think scientifically about and invent a product or service to try and solve the issue.	Energy Audit	Students will explore the ways that we all use energy and will investigate their energy choices and see how much energy they use per day doing an energy audit.
6	Art in Nature	Students will learn about sustainability in art and explore the works of artists such as Andy Goldsworthy.	Your Environmental Impact	Students will apply their knowledge on energy and their audits to the world and explore the ways their energy choices affect the planet.
7	Ecology	Students will learn ecological relationships through various activities such as making soil and making a large food web as a class.	Eat Well!	Students will learn about nutrition through the MyPlate nutrition tool and will understand how their food breaks down into fats, carbohydrates, proteins, minerals, vitamins, and water. They will also understand how to read nutrition labels.
8	Evolution	Students will take what they have learned about ecological relationships and build on this with activities surrounding adaptations, natural selection, and evolution.	Healthy Earth, Active People	Students will learn why time outdoor playing is important and will explore new activities that they can do outside.
9	Habitats Around the World	Students will learn about the different habitats around the world and cover a basic overview of the main habitats and biomes.	Healthy Communities	Students will learn ways that communities come together to practice healthy habits. Students will also apply knowledge about LNT and pollution to talk about what a healthy community might look like.
10	What is Your Habitat?	Students will select one specific habitat to learn about more in depth. The activities will range depending on the habitat that the group is delving into. Students will present what they learned to the class.	Camping basics	Students will learn the basics of camping and explore different camping and outdoor skills.
11	Food Production	Students will apply knowledge of food webs to modern-day farming. They will follow the journey of a hamburger and go back to the source of each component that makes up a hamburger.	Map reading and Orienteering	Students will try their hand at Map reading and Orienteering.
12	Water Cycle	Students will interactively explore the water cycle. Younger students will cover the basics, while older students will delve into topics such as water scarcity.	Project (Week 1)	Students begin working on month-long projects. In groups, they will pick an environmental issue or a topic of interest, and work in their teams throughout the remaining weeks to create a product or service that will solve or bring awareness to this issue.
13	Weathering and Erosion	Students will understand how the water cycle and different natural forces change the make-up of land.	Project (Week 2)	Work in teams on projects + team-building activities.
14	Rock Cycle	Students will explore the rock cycle. Younger students will cover the basics, while older students will explore Fossils and Oxidation weathering.	Project (Week 3)	Work in teams on projects + team-building activities.
15	Plate Tectonics	Students will understand plate tectonics and learn how scientists are able to see how Earth has changed over time.	Project Presentations and Closing Activities	Final day of GGO programming—project presentations with a day of fun outdoor activities and a close-out reflection of fun memories that were had throughout the semester.

Month	Field trip	Augmented in-class lessons	Field trip	Augmented in-class lessons
1	Urban Hike in Forest Park (St. Louis, MO)	(1) LNT, (2) Green City, (3) Habitats Around the World (4) Healthy Earth, Active People	Rock Climbing (St. Louis, MO)	(1) Rock Cycle, (2) Healthy Earth, Active People
2	Overnight Camping Trip (Giant City State Park, IL)	(1) LNT, (2) Art in Nature, (3) Ecology, (4) Habitats Around the World, (5) Healthy Earth, Active People	Science Museum (St. Louis, MO)	(1) Scientists (2) Ecology (3) Rock Cycle, (4) Plate Tectonics, (5) Weather vs. Climate
3	Volunteering at Urban Farm (St. Louis, MO)	(1) Ecology (2) Food Production (3) Eat Well!	Canoe Trip Down the Mississippi River	(1) LNT, (2) Water Cycle, (3) Weathering and Erosion, (4) Pollution, and (5) Healthy Earth, Active People
4	Cave Trip (Sullivan, MO)	(1) LNT, (2) Habitats Around the World, (3) Weathering and Erosion, (4) Rock Cycle	Overnight Camping Trip (Giant City State Park, IL)	(1) LNT, (2) Art in Nature, (3) Ecology, (4) Habitats Around the World, (5) Healthy Earth, Active People

GGO, Gateway to the Great Outdoors; LNT, Leave No Trace.

The average duration of the intervention was 15 weeks. Data from the SLPS students were collected through a pre-intervention survey in January 2019 and a post-intervention survey in May 2019 (Spring semester 2019). Qualitative data, from the focus groups, were collected in April 2019. Data from the SLPS schoolteachers and NBE mentors were collected through a post-intervention survey in May 2019. Washington University in St. Louis's Institutional Review Board deemed this study as exempt from review since the data were anonymous.

### Quantitative analysis

The study used a pre-test–post-test study design to evaluate HRQoL and STEM capacity during the NBE intervention. A self-administered 22-item survey was distributed to the SLPS students before participating in the intervention (pre-intervention) and after completing the intervention (post-intervention). The survey collected information about age, gender, race, and past STEM and nature contact experiences before entering the intervention. The pre-intervention–post-intervention survey consisted of five widely used, validated, HRQoL domains (physical health functioning, emotional health functioning, school functioning, social functioning, and family functioning)^51–53^ and six STEM-capacity domains (leadership, teamwork, science relevance, sustainability relevance, STEM self-efficacy, and science interest). The STEM-capacity instrument was informed by current STEM education literature as well as input from the SLPS teachers.^34,54–59^ Responses to the HRQoL domains were assessed using 5-point Likert-type scales ranging from 1 to 5, and responses to the STEM-capacity domains were assessed using a 3-point Likert-type scale ranging from 1 to 3. Lower scores indicated better HRQoL and STEM capacity.

HRQoL and STEM-capacity items were reverse-scored so that higher scores indicated better HRQoL and STEM capacity, respectively. An overall HRQoL score and an overall STEM-capacity score were determined by summing the respected domain scores. Pre-intervention–post-intervention differences in STEM capacity and HRQoL were assessed using independent t-tests. Linear regression models were used to assess each HRQoL and STEM-capacity domain score by age, gender, and duration in the intervention.

### Qualitative analysis

To provide additional qualitative data, a focus group was held for each classroom participating in the NBE intervention. These focus groups were incorporated into the intervention to provide a more holistic insight into the NBE intervention's impact on the HRQoL and STEM capacity.^17,60^ The study completed 10 focus groups to collect qualitative data on the SLPS students' engagement, learning outcomes, and experience with the NBE intervention. Focus groups composed of 10–25 children who participated in the NBE intervention. The moderator posed predetermined questions to participants, added questions to probe answers, and assured that the discussion remained on the subject of interest. The focus group discussions lasted ∼30 min. Focus group discussions were taped and transcribed.

We used Braun and Clarke's method to analyze the major themes appearing in the focus groups.^61,62^ We identified the themes as they appeared, while reviewing the data, rather than analysis based on an existing theoretical framework. First, three trained reviewers conducted an initial review of the transcribed focus groups to familiarize themselves. Then, the reviewers independently generated potential themes to code. The three reviewers evaluated the potential themes and reached consensus on a final set of themes. One reviewer then coded the transcribed focus groups based on the finalized themes. Quotes that contained multiple themes were coded with more than one theme. The distribution of themes per focus group was determined and the reviewers selected representative quotes for each theme.

### NBE mentors' and schoolteachers' perceptions analysis

After completion of the course NBE mentors and schoolteachers completed a 17-item post-intervention survey. The survey collected information on their perceptions of the NBE intervention impact on the SLPS students. The survey administered to the NBE mentors and the schoolteachers was summarized with univariate analyses. For the open ended section of the post-survey, we used Braun and Clarke's method described above to identify the major themes of the interviews.^61,62^

## Results

### Participant characteristics

A total of 122 SLPS students participated in the NBE intervention during the Spring semester 2019. Children's ages ranged from 10 to 15 years. Each participant completed the pre-intervention survey and all but two completed the post-intervention survey. Table 2 displays the demographic distribution of the participants.

**Table 2.** Demographic Distribution of St. Louis Public School Student Participants (*n*=122)

	Total	School 1	School 2	School 3
Grade levels participating		Sixth, seventh, eighth	Sixth	Fifth
Average age (SD)	11.9 (1.0)	12.9 (0.9)	11.6 (0.5)	11.1 (0.9)
Average semesters in program (SD)	1.7 (1.1)	2.6 (1.5)	1 (0.0)	2 (0.0)
Gender				
M	61	19	29	13
F	61	18	32	11
Ethnicity				
Non-Hispanic Black	102	31	48	23
Hispanic Black	16	5	10	1
Other^a^	4	1	3	0
Total	122	37	61	24

^a^ Other includes one white Hispanic, one white non-Hispanic, one American Indian or Alaska Native, and one Asian.

**FIG. 2.**
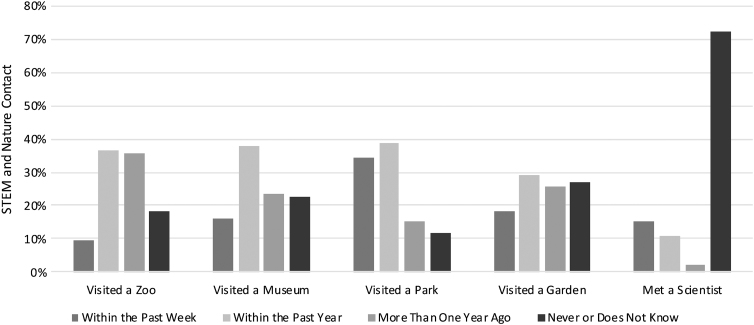
SLPS students' STEM and nature contact before the education intervention (*n*=68). SLPS, St. Louis Public School.

During the Spring 2019 semester, 68 of the 122 children participated in the intervention for the first time. In the year before the intervention, of these 68 participants, 54% had not visited a zoo, 46% had not visited a museum, 27% had not visited a park, 53% had not visited a garden, and 74% had not met a scientist (Fig. 2).

### Health-related quality of life

We saw significant improvements in all HRQoL domain (physical health functioning, emotional health functioning, school functioning, social functioning, family functioning, and overall HRQoL) mean and median scores from pre-intervention to post-intervention (all *p*<0.05). We further tested the interactions between age, gender, and duration in the intervention with the pre-intervention to post-intervention scores for each HRQoL domain. There was no significant interaction between gender and duration in the intervention with the pre-intervention and post-intervention mean and median HRQoL domain scores (all *p*>0.05). For age, interactions were significant for the physical health domain (*p*=0.019), social functioning domain (*p*=0.005), and overall HRQoL domain (*p*=0.001). Therefore, the results presented are stratified by age in Table 3.

**Table 3.** Health-Related Quality-of-Life Scores Pre-Intervention and Post-Intervention, Stratified by Age (*n*=120)

HRQoL	Overall		Eleven and under		Twelve		Thirteen and older		Interaction p-value
	Pre-intervention score (SD)	Post-intervention score (SD)	Pre-intervention score (SD)	Post-intervention score (SD)	Pre-intervention score (SD)	Post-intervention score (SD)	Pre-intervention score (SD)	Post-intervention score (SD)	
Physical health functioning	3.3 (1.4)	4.5 (1.2)	3.7 (1.5)	4.0 (1.4)	3.1 (1.4)	4.5 (1.1)	3.1 (1.4)	4.4 (1.1)	**0.019**
Emotional health functioning	2.4 (1.2)	4.1 (0.9)	2.6 (1.7)	4.0 (1.0)	2.3 (0.9)	4.2 (0.9)	2.2 (1.2)	4.2 (0.7)	0.206
School functioning	3.1 (1.3)	4.2 (0.9)	3.5 (1.3)	4.2 (0.9)	2.9 (1.3)	4.2 (1.0)	3.1 (1.3)	4.3 (0.8)	0.141
Social functioning	2.6 (1.2)	4.1 (0.1)	3.2 (1.2)	3.9 (1.2)	2.42 (1.1)	4.2 (1.0)	2.2 (1.2)	4.0 (1.1)	**0.005**
Family functioning	2.1 (1.3)	3.0 (1.6)	2.2 (1.4)	2.9 (1.5)	1.9 (1.3)	2.9 (1.6)	2.2 (1.2)	3.1 (1.5)	0.829
Overall HRQoL	13.5 (3.7)	19.9 (2.8)	15.2 (4.2)	18.8 (3.1)	12.6 (3.1)	19.9 (2.9)	12.8 (3.4)	19.9 (2.8)	**0.001**

Bold type indicates statistically significance.

HRQoL, health-related quality of life

Age was not a significant interaction variable for emotional health functioning, school functioning, or family functioning HRQoL domains (all *p*>0.10). The mean emotional health functioning HRQoL domain score increased from 2.4 in the pre-intervention survey to 4.1 in the post-intervention survey (*p*<0.001). The mean school functioning HRQoL domain score increased from 3.1 in the pre-intervention to 4.2 in the post-intervention (*p*<0.001). The family functioning HRQoL domain mean score rose from 2.1 to 2.9 after the NBE intervention (*p*=0.001).

### STEM capacity

We tested the interactions between age, gender, and duration in the intervention with the pre-intervention to post-intervention scores for each STEM-capacity domain. There was no significant interaction between age and gender with the pre-intervention and post-intervention mean and median domain scores (all *p*>0.05). For duration in the intervention, there were significant interactions for all STEM-capacity domain scores. Therefore, we present the results stratified by duration in the intervention in Table 4. Each STEM-capacity domain (leadership, teamwork, science relevance, sustainability relevance, STEM self-efficacy, science interest, and overall STEM-capacity) mean and median score significantly increased from pre-intervention to post-intervention (all *p*<0.05).

**Table 4.** Science, Technology, Engineering, and Math-Capacity Scores Pre-Intervention and Post-Intervention, Stratified by Learning Duration (*n*=120)

STEM capacity	Overall		First semester		Two or more semesters		p-Value of interaction
	Pre-intervention score (SD)	Post-intervention score (SD)	Pre-intervention score (SD)	Post-intervention score (SD)	Pre-intervention score (SD)	Post-intervention score (SD)	
Leadership	1.3 (0.8)	1.7 (0.5)	1.1 (0.8)	1.7 (0.4)	1.6 (0.7)	1.8 (0.5)	**0.004**
Teamwork	1.3 (0.7)	1.6 (0.5)	1.0 (0.7)	1.5 (0.6)	1.5 (0.6)	1.7 (0.5)	**0.023**
Science relevance	1.4 (0.7)	1.7 (0.5)	1.2 (0.7)	1.7 (0.5)	1.6 (0.6)	1.8 (0.4)	**0.026**
Sustainability relevance	0.9 (0.8)	1.8 (0.5)	0.6 (0.8)	1.8 (0.4)	1.3 (0.7)	1.7 (0.5)	**<0.001**
STEM self-efficacy	1.0 (0.8)	1.7 (0.5)	0.8 (0.8)	1.7 (0.5)	1.2 (0.8)	1.7 (0.6)	**0.001**
Science interest	1.2 (0.7)	1.5 (0.6)	1.1 (0.7)	1.6 (0.5)	1.4 (0.7)	1.4 (0.7)	**0.005**
Overall STEM capacity	7.0 (2.8)	10.1 (1.7)	5.8 (2.6)	10.1 (1.6)	8.5 (2.1)	10.0 (1.7)	**<0.001**

Bold type indicates statistically significance.

STEM, science, technology, engineering, and math.

### Qualitative results

**FIG. 3.**
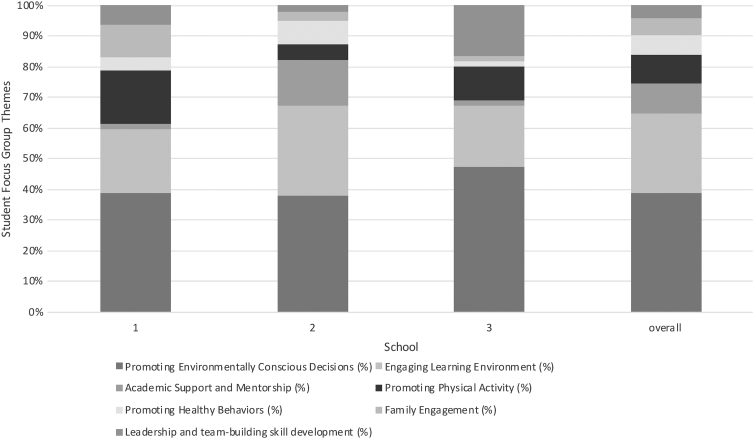
Theme distribution from SLPS student focus groups by school.

The thematic analysis of the focus groups revealed seven major themes: Engaging Learning Environment (26% of the themes), Promoting Environmentally Conscious Decisions (39%), Family Engagement (6%), Promoting Healthy Behaviors (6%), Promoting Physical Activity (9%), Leadership and Team Building Skill Development (4%), and Academic Support and Mentorship (10%). Figure 3 shows the distributions of the themes by school and overall. Table 5 displays each theme with representative quotes from the focus groups.

**Table 5.** Examples of Quotes from St. Louis Public School Student Focus Groups by Theme

Theme	Sample quote
Engaging Learning Environment	
	I would do it again because it was educational. I liked all the field trips, the activities. They were just so much fun. I enjoyed this program and I'd love to do it again
	[Before the program] we used to do these [lessons] in our workbooks. Just doing work, being bored… When they came we did activities and stuff like that. They made learning fun.
	We were exploring things, and we got to see things that we don't see, usually
	I like how the field trips relate to what they talk about
Promoting Environmentally Conscious Decisions	
	I started recycling more and stopped wasting food
	I started recycling more and I started reusing water bottles and stuff
	We live in environment, so we should, like, take care of our environment, like pick up trash and stuff
Family Engagement	
	My momma she was trying to plant this plant and I showed her how to do it, how to prepare, how to plant those… I showed her how to water and stuff to grow it
	Like we have soda cans in those little plastic thingies, the rings and stuff like that. My mom doesn't buy those anymore… She buys the one with the cardboard, and then she reuses it to make art projects and stuff like that. Then metal straws. We use metal straws [now].
	I told her [my mom] an interesting fact we learned, ‘cause I like to learn facts ‘cause it makes me seem smarter than her, so I just told her
Promoting Healthy Behaviors	
	We learned the difference between vegetables and fruit and that they have… calories and fat
	I learned to read the nutrition thing on the back of foods to see how much calories you are eating
	They were talking about calories and some good foods that we can eat to keep us healthy
Promoting Physical Activity	
	To go to the store, instead of—if it's a short distance we just walk instead of taking the car all the time
	I would say put down the screens and go outside and enjoy what's around you instead of staring at your screen
Leadership and Team-Building Skill Development	
	I learned how to work with people, and the thing is you could actually learn from
	We had to learn about a specific topic and then teach sixth-graders about the topic we learnt about
Academic Support and Mentorship	
	They were nice, and they helped us with our work, and if we didn't want to do it, they would talk to us and see what happened or a bad day or stuff like that
	They were sweet and respectful to us, and… every time we need their help, they would always come and help us when we needed it.
	Something that I liked most about the… program was that they helped us understand things that if we didn't get it, they would help us slowly figure it out and give us chances to figure it out ourselves before they just told us what the answer is
	If we have something to do and they'll actually help us make it, turn it into a fun lesson so that we can understand

### NBE mentor and schoolteacher perceptions of impact

A total of 49 NBE mentors and 5 schoolteachers completed a post-survey on their perceptions of the NBE impacts on the SLPS students. Every NBE mentor and SLPS teacher indicated that the NBE intervention was beneficial for the SLPS students. Answers to why the NBE intervention was beneficial for the children varied; however, four major themes appeared: *(1) Enriched Educational Experiences, (2) Increased Environmental Awareness, (3) Novel Experiences,* and *(4) Behavioral Change*. Table 6 displays each theme with representative quotes.

**Table 6.** Examples of St. Louis Public School Teacher and Nature-Based Education Mentor Responses to “Why Was the Nature-Based Education Intervention Beneficial to Your Students?“

Theme	Sample quote	School	Grade working with	Position
Enriched Educational Experience				
	NBE makes students love science and look forward to practicing science, reading, and writing. It allows students to think outside of the classroom.	1	8	SLPS teacher
	It gives the students to learn in a fun and interactive way.	2	6	NBE mentor
Increased Environmental Awareness				
	NBE both teaches them environmental information that they may not be getting in their normal classes, makes them think about the world in a different way, and gives them a break from normal schoolwork in the day.	2	6	NBE mentor
	The students were able to learn about the environment and sustainability. This is information that they otherwise might not receive in school, even though it is very important to learn about.	1	6	NBE mentor
	The students were able to learn about the environment and sustainability. This is information that they otherwise might not receive in school, even though it is very important to learn about.	2	6	NBE mentor
	I think it's very beneficial to the students. On trips, they not only make meaningful experiences, but also develop connection to the outdoors that no textbook can provide.	3	5	NBE mentor
Novel Experiences				
	Yes, I think GGO is beneficial to the students because they got to see, try, and do things they've never done before… It's a good experience for the students and the teachers.	3	5	SLPS teacher
	Most of the time what we teach them or when we go on trips, they are doing things that they've never done before and seem genuinely interested.	2	6	NBE mentor
Behavioral Change and Students Reaching Full Potential				
	The students have a lot to say once you get them motivated. They are very smart, and this organization helps them realize their true potentials.	2	6	NBE mentor
	I can definitely see improvements in the students' behavior when learning inside the classroom and when learning outside during field trips.	1	7	NBE Mentor
	A great number of students talk about how much they learn and tell me how much the lessons have changed their perspectives.	2	6	NBE mentor

NBE, nature-based education; SLPS, St. Louis Public School.

**FIG. 4.**
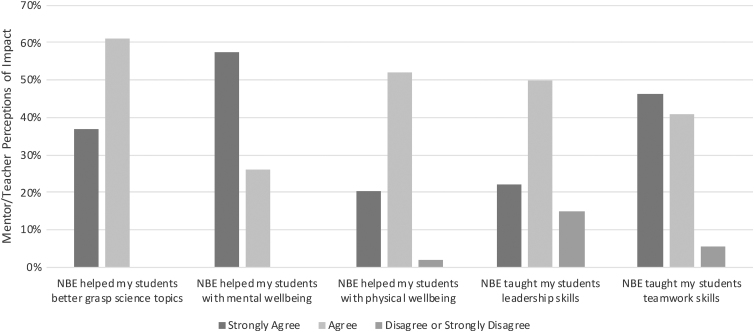
NBE mentor and school teacher perception of impact.

Figure 4 shows the NBE mentors' and schoolteachers' perceptions of NBE's impact on the SLPS students. Approximately 98% of NBE mentors and schoolteachers agreed or strongly agreed that NBE helped the SLPS students better grasp scientific topics, 83% agreed that NBE helped the SLPS students' mental well-being, 72% agreed that NBE helped with the SLPS students' physical well-being, 72% believed that NBE taught the SLPS students leadership skills, and 87% agreed or strongly agreed that NBE taught the SLPS students teamwork skills.

## Discussion

This study evaluated an NBE intervention's influence on the HRQoL and STEM capacity for low-income, urban, black and Hispanic children. We observed significantly higher scores in every HRQoL and STEM-capacity domain after the completion of the intervention. These findings are consistent with a growing body of evidence that nature contact and environmental education improve health outcomes and scientific engagement^29,34,63,64^ and supports future research with stronger study designs addressing NBE as a sustainable method to reducing health and educational disparities for low-income, urban, black and Hispanic youth.

We found significant interactions with age and the improvement in the physical health functioning, social functioning, and overall HRQoL domain scores; older children (12–15 years of age) had larger improvements from the intervention than younger children (10–11 years of age). The older children had lower physical health functioning HRQoL pre-intervention scores than the younger children. This could be caused by the well-documented decline in physical activity for children during the transition from elementary to middle school.^65^ In addition, there is a large body of evidence that suggests children experience increased social problems and decreased HRQoL before and during middle school.^66,67^ This might account for the lower pretest scores we observed in older children for social functioning and overall HRQoL domains. After the intervention, all three age brackets had approximately the same social functioning and overall HRQoL domain score. Similarly, a 3-year cohort study found increased time spent outdoors significantly was associated with increased physical activity and decreased obesity for older children (10 to 12 years of age), but the association was not observed in younger (5–6 years of age) children.^21^

We also found significant improvements in all STEM-capacity domain scores, confirming past results.^43,68^ Our study also found significant interactions with the duration a student participated in the intervention and the differences between the pre-intervention and post-intervention STEM-capacity scores, and that outdoor classes increased long-term knowledge retention for students. Together, these results support the idea that multiple years of NBE could increase its benefits.

There is little agreement on how to define exposure for both nature contact and environmental education. Exposure to nature includes duration, frequency, and magnitude or intensity of exposures. Currently, the majority of nature-contact exposure science studies focus on the magnitude and frequency of nature contact, not duration.^29^ Magnitude or intensity of exposure is also poorly quantified, but clearly, there is a range of intensities from a single plant in a hospital room to multiday wilderness camping trips. One study of adults in England found that spending at least 120 min a week in nature significantly increases the likelihood of reporting good HRQoL compared to those who had no nature contact.^69^ However, additional research is required to better understand the dose–response relationship between NBE and nature contact with beneficial outcomes.^29^

As in past studies, our thematic analysis of focus group responses revealed that NBE promotes an engaging learning environment, promotes environmentally conscious decisions, engages family, promotes healthy behaviors and physical activity, as well as develops leadership and team building skills.^29,34^ One study reported that NBE was a worthwhile addition to the middle school curriculum by promoting physical activity, teaching leadership and teamwork, promoting environmental stewardship, and offering relevant and memorable learning experiences.^70^ Our results are also consistent with these findings.

This study has several limitations. First, this developmental study had a modest sample size and duration. Second, the study population consisted of children from an urban public school system in St. Louis, Missouri, potentially reducing generalizability. Third, this study did not include a control group. Finally, our STEM-capacity instrument has not been validated, although it was based on research on the educational outcomes of environmental education.

Future research should use improved study design with the inclusion of control groups to strengthen estimates of the effects of this NBE on children's HRQoL and STEM capacity and consider longer follow-up of health and academic outcomes. In addition, results from our focus groups suggest several potential moderators of the effects of NBE, such as stressful life events. Measures of these stressors could strengthen future evaluation studies. Surveys or focus groups of the participating children's guardians on their perceptions of the NBE intervention's impact would also strengthen future studies. In the United States, educational and health disparities are often rooted in structural inequities, and additional research is needed, examining effects of the NBE in different school settings, as well as research comparing other forms of NBE (e.g., summer camps, educational programs at botanical gardens, and outdoor adventure education). The evaluation of NBE interventions in nonminority schools and in high-income public and private schools could help determine to what extent NBE reduces health and educational disparities. Future research should also explore dose–response relationships for NBE by examining interventions of different durations (e.g., 1 week, 1 month, and 1 year).

## Conclusion

Our study found that low-income, urban, black and Hispanic children significantly improved their HRQoL and STEM capacity after participating in an NBE intervention in St. Louis, Missouri. These results support the need for more rigorous examination of the potential effects of this NBE intervention as a low-cost and sustainable method for improving HRQoL and STEM capacity for students at low-income schools. Larger studies with the inclusion of objective measures of behavioral and educational outcomes, a control group of students not participating in an NBE intervention, and a high-income population participating in NBE intervention will better investigate how NBE would be a feasible solution for reducing health and educational disparities.

## Abbreviations Used

GGO: Gateway to the Great Outdoors
HRQoL: health-related quality of life
LNT: Leave No Trace
NBE: nature-based education
SLPS: St. Louis Public School
STEM: science, technology, engineering, and math
